# The relationship between perfectionism and depression among college students: a multiple mediation mechanism based on coping styles, loneliness, and self-esteem

**DOI:** 10.3389/fpsyg.2026.1787420

**Published:** 2026-03-31

**Authors:** Yonghua Liu, Zhongjun Sun

**Affiliations:** 1 School of Teacher Education, Xinyang Normal University, Xinyang, Henan, China; 2 School of Physical Education and Health, Zhaoqing University, Zhaoqing, Guangdong, China

**Keywords:** coping style, depression, loneliness, perfectionism, self-esteem

## Abstract

**Background:**

This study examined the associations between perfectionism and depression among college students and explored the mediating roles of coping style, loneliness, and self-esteem in these relationships.

**Methods:**

Using validated instruments, we assessed perfectionism, coping style, loneliness, self-esteem, and depression in a sample of college students. We then tested a multiple mediation model using Partial Least Squares Structural Equation Modeling (PLS-SEM).

**Results:**

The results showed that different forms of perfectionism were associated with depression in distinct ways. Coping style, loneliness, and self-esteem each played significant mediating roles in these associations.

**Discussion:**

These findings deepen understanding of how psychological resources and emotional experiences are involved in the relationship between perfectionism and depression among college students. The study also provides practical implications for psychological support and intervention in university settings.

## 1 Introduction

The university period plays a key role in an individual's personal growth and social integration. It is not only a time for accumulating professional knowledge and skills but also an important moment for shaping values and worldviews. However, depression has a severe impact on college students, manifesting in symptoms such as emotional low mood, loss of interest, lack of appetite, and insomnia. In severe cases, it may even lead to self-neglect and confusion about future development, further hindering academic and career success (Fang and Wang, 2024). Among the various psychological health issues affecting Chinese college students, depression has a high incidence rate of 20.8%, ranking second (Fu et al., 2020). Data indicates that approximately 9.8% of Chinese college students experience varying degrees of depressive symptoms (Han et al., 2025). The onset of depression is influenced by multiple factors, including external and internal factors. External factors primarily refer to stressors in the environment, such as academic pressure, career planning, and social adaptation, which may lead to emotional distress. For college students, academic pressure and interpersonal relationship issues are common external factors that often trigger anxiety and depression. Lack of social support and feelings of loneliness also exacerbate psychological health problems (Hager et al., 2022). Internal factors mainly involve an individual's psychological traits, such as excessively high self-expectations, excessive worry about failure, coping strategies, and negative self-assessment of abilities and value. These factors may cause individuals to experience excessive anxiety, helplessness, or self-doubt when facing challenges, thereby increasing the risk of emotional distress (Liu et al., 2024; Wang et al., 2024; Wei et al., 2021). Therefore, identifying the key influences and mechanisms behind depression in college students is vital for creating targeted and effective intervention approaches.

Although perfectionism has been recognized as a significant predictor of depression, especially within the college student population, excessive self-expectations and the pursuit of perfection can easily lead to emotional distress and the onset of depressive symptoms (Wei et al., 2021). However, existing research has certain limitations. On one hand, most studies analyze perfectionism using overall scores or simple dimensions, failing to explore the differences between various types of perfectionism. On the other hand, current research lacks attempt to integrate key psychological factors, such as behavioral regulation mechanisms (e.g., coping styles), social-emotional experiences (e.g., loneliness), and self-evaluation (e.g., self-esteem), into a unified model. This study therefore builds a multiple mediation model to examine how coping styles, loneliness, and self-esteem (SE) mediate the depression process, which is influenced by different aspects of perfectionism. This model helps uncover more complex psychological mechanisms and provides new theoretical support and practical directions for understanding the roots of depression among Chinese college students and possible intervention methods.

## 2 Theoretical framework and hypotheses

### 2.1 Theoretical basis

Social Cognitive Theory primarily emphasizes the interaction between an individual's behavior, cognition, and environment (Chou et al., 2019). The theory proposes that an individual's actions are determined by both external influences and the management of their cognitive processes. Specifically, individuals form an understanding of their abilities by observing the behavior of others and its consequences, and these perceptions are then translated into behavioral choices and adjustments (Chou et al., 2019). Research has found that, among college students, perfectionism as a stable personality trait may influence how students perceive, interpret, and respond to stress events, thereby indirectly affecting the onset of depressive emotions (Edmonds et al., 2022). In particular, perfectionists may tend to set excessively high self-expectations and worry about failure. This excessive focus on personal performance makes them more susceptible to stress and likely to adopt negative coping style (NCS), ultimately exacerbating emotional issues (Wei et al., 2021).

The Stress-Vulnerability Model further reveals an individual's psychological vulnerability in the face of stress and its impact on emotional regulation. This model posits that an individual's psychological vulnerability (e.g., NCS, loneliness, low SE) could intensify the adverse effects of external stressors on mental health (Demke, 2022). Specifically, within the college student population, perfectionists tend to exhibit higher stress perception and lower self-awareness, which increases their risk of developing depressive emotions. Research has indicated that NCS, elevated levels of loneliness, and low SE are significant predictors of depression (Tian et al., 2025). When facing academic pressure and social challenges, perfectionists are likely to experience greater feelings of isolation and self-neglect, further exacerbating their depressive symptoms.

By combining the Social Cognitive Theory with the Stress-Vulnerability Model, we find that these two theories complement each other in this study, collectively explaining how perfectionism influences depression among college students through mediating variables such as coping styles, loneliness, and SE. Social Cognitive Theory emphasizes how individuals adjust their behavior and emotions through self-cognition, while the Stress-Vulnerability Model adds the role of individual psychological vulnerability in stress, pointing out that NCS, loneliness, and low SE exacerbate depressive emotions. This integration highlights the dynamic balance between psychological factors and environmental interactions, explaining the interplay of behavior, emotions, and psychological vulnerability in the onset of depressive symptoms, while offering a theoretical basis for formulating targeted psychological intervention strategies.

### 2.2 Hypotheses

#### 2.2.1 Perfectionism and depression

In recent years, perfectionism has gained widespread attention as an important personality trait influencing college students' mental health. Perfectionism has two dimensions: adaptive and maladaptive, providing a more nuanced perspective on its relationship with depression (Zhang et al., 2021). Maladaptive perfectionism (MP) is characterized by sensitivity to mistakes, excessive concern about others' evaluations, and self-neglect, which has been shown to be positively correlated with depressive symptoms (Fang and Wang, 2024; Wei et al., 2021). Research by Wei et al. (2021) on perfectionism and depression among Chinese college students found that individuals with MP are more prone to catastrophic thinking and negative thoughts, which amplify the impact of failure and increase the likelihood of experiencing depressive emotions. Additionally, Hewitt et al. (2022) further found that maladaptive perfectionists are more likely to experience shame and helplessness due to evaluative anxiety, which triggers depression. In contrast, adaptive perfectionism (AP) emphasizes self-directed high standards and positive achievement motivation, which is significantly negatively correlated with depression (Olmedilla et al., 2022; Jeong and Kim, 2021). Jeong and Kim (2021) found that individuals with AP tend to have higher self-efficacy and goal alignment, and they are better at regulating emotions and making positive attributions when facing stressful events, which reduces the risk of depression. Furthermore, from the perspective of social functional adaptability, these individuals demonstrate a strong sense of responsibility and execution in interpersonal relationships. This helping to establish a good support system, improving their sense of belonging and SE, and improve psychological resilience, thus buffering the impact of negative emotions (Milicev et al., 2023). In summary, MP is a key risk factor for depression in college students, while AP may, to some extent, act as a protective factor by enhancing individuals' internal resources and external support networks.

#### 2.2.2 The potential mediating role of coping styles

Coping styles refer to the cognitive and behavioral strategies individuals adopt when facing stress or challenges, aimed at regulating emotions, alleviating psychological burdens, and adapting to the environment. These strategies are typically categorized into positive coping style (PCS) and negative coping style (NCS) (Quchani, 2023). PCS includes problem-solving, seeking support, and cognitive reappraisal, which help with emotional regulation and stress management. In contrast, NCS such as avoidance, suppression, and self-blame often exacerbate negative emotions and reduce adaptability (Lu et al., 2024). Dong et al. (2024) reveals that students who employ PCS generally exhibit lower levels of depression because they are better at utilizing external and internal psychological resources to alleviate stress and negative emotions. Conversely, students who tend to use NCS are more likely to get trapped in emotional difficulties and lack effective means of regulation, which increases their risk of depression (Zhang and Hu, 2024). Coping styles, to some extent, determine an individual's cognitive appraisal and coping effectiveness in response to stressful events, making them an important psychological mechanism influencing the occurrence of depression. Additionally, perfectionism also has a significant impact on coping styles (Siah et al., 2022; Xu et al., 2024). Research by Samfira and Palos (2021) indicates that AP is closely related to PCS, with individuals showing higher motivation and actively adopting problem-solving approaches to achieve their goals. In contrast, individuals with MP are more likely to experience external pressures and tend to use NCS such as avoidance and self-blame, which can further intensify psychological burdens and depressive experiences (Montano, 2025). In summary, different coping styles may function as mediating factors in the relationship between multiple dimensions of perfectionism and depression among college students.

#### 2.2.3 The potential mediating role of loneliness

Loneliness is a negative emotional experience triggered by subjective feelings of social isolation. It not only manifests as a lack of social connections but also involves the perception of insufficient emotional support and a sense of belonging at the psychological level (Cipolletta et al., 2025). Loneliness is common among college students and is often highly correlated with mental health issues (Yu et al., 2022; Hee and Hyun, 2023). Research by Hee and Hyun (2023) found that loneliness can trigger an individual's sensitivity to social threats, leading to negative interpretative biases regarding others' behavior, which exacerbates social avoidance behaviors and depressive symptoms. Similarly, Chang and Yang (2024) in their study on loneliness and depression in both China and the United States found that loneliness weakens an individual's ability to cope with stress, making them more susceptible to feelings of helplessness when facing challenges such as academics and employment, ultimately triggering or exacerbating depressive emotions. At the personality trait level, perfectionism is widely regarded as an important variable influencing loneliness (Shafiq et al., 2024). Adaptive perfectionists tend to have higher self-efficacy and achievement experiences, which help them establish stable social connections and a sense of belonging (Visvalingam et al., 2024; He and Ding, 2025). He and Ding (2025), in their research on emotional and motivational factors among Chinese college students, pointed out that AP may reduce loneliness by enhancing achievement motivation and goal-oriented behaviors, promoting positive development in areas such as academics and social interactions. In contrast, MP significantly increases loneliness (Niels-Kessels et al., 2025; Blynova et al., 2021). Blynova et al. (2021) found that maladaptive perfectionists, due to excessive concern about others' expectations and evaluations, tend to experience subjective feelings of isolation and misunderstanding, and are more prone to self-criticism and social avoidance behaviors, leading to a long-term loneliness-depression vicious cycle. Furthermore, research by Rnic et al. (2021), based on the Perfectionism-Social Disconnection Model, suggests that individuals with perfectionist traits and a focus on self-presentation are more likely to develop depressive symptoms when they experience social disconnection, especially when feeling socially hopeless and lonely. Therefore, this study posits that loneliness can act as a potential mediating variable to explain the mechanisms through which different dimensions of perfectionism influence depression among college students.

#### 2.2.4 The potential mediating role of self-esteem

Self-esteem (SE) represents a person's overall judgment of their personal value and abilities, reflecting their subjective sense of self-identity, behavioral competence, and social adaptability (Burkitt, 2024). College students experience a crucial stage of rapid physical and psychological growth, during which SE gradually forms and becomes more complex, exerting a profound impact on mental health (Doyle and Catling, 2022). Research by Jiang and Zhang (2023) reported a positive association between SE and constructive emotion regulation strategies, which effectively buffer the negative effects of external stressors, thereby reducing depression levels. Lei et al. (2024), in their survey of 1,265 Chinese students based on cognitive vulnerability and the scar model, found that low SE is closely associated with cognitive biases such as negative attribution and self-neglect, which foster a pessimistic psychological pattern that makes college students more prone to feelings of helplessness and depression. On the other hand, perfectionism is one of the key personality traits that influence the formation and change of SE among college students (Khossousi et al., 2024). AP is typically characterized by proactivity, self-motivation, and the pursuit of achievement. The sense of goal-directedness and efficacy fostered by AP helps individuals form a stable sense of self-worth, thereby enhancing SE (Burkitt, 2024; Bal and Arikan, 2023). Raedeke et al. (2021) found that these individuals are more likely to experience self-satisfaction after achieving their goals and maintain positive cognition in social interactions, establishing a healthy system of self-acceptance and self-evaluation. In contrast, MP emphasizes compliance with external standards and fear of failure, often accompanied by self-neglect and excessive self-criticism, which undermines SE (Doyle and Catling, 2022; Fearn et al., 2022). Doyle and Catling (2022) highlighted those maladaptive perfectionists, even after achieving success, struggle to acknowledge their accomplishments and fall into a continuous cycle of low self-evaluation, exhibiting significant psychological vulnerability. Furthermore, research by Chai et al. (2020) on perfectionism and depressive symptoms among Chinese college students found that individuals with AP often alleviate depressive emotions indirectly through the establishment of positive self-identity and high levels of SE, while maladaptive perfectionists, due to prolonged self-neglect, are prone to SE damage, which exacerbates their depression levels. Therefore, this study posits that SE may play a potential mediating role in the pathway through which adaptive and MP influences depression among college students.

## 3 This study

**Figure 1:**
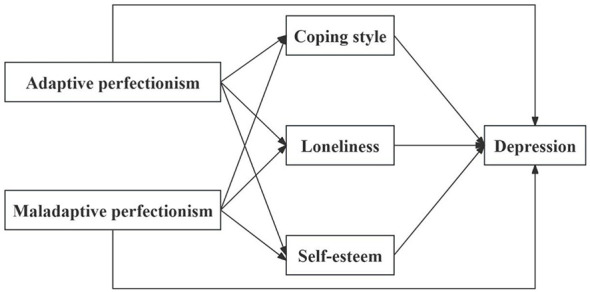
Hypothetical model.

Building on previous research, this study systematically explores the impact of different dimensions of perfectionism on depression among college students, integrating Social Cognitive Theory and the Stress-Vulnerability Model. Additionally, to more precisely analyze how perfectionism affects the onset and development of depression, this study is the first to comprehensively validate the mediating roles of coping styles, loneliness, and SE in this relationship. A multiple mediation model (Figure 1) was constructed, and the following hypotheses were tested:

H1: Adaptive perfectionism significantly negatively predicts depression among college students.

H2: Maladaptive perfectionism significantly positively predicts depression among college students.

H3: Coping styles mediate the relationship between adaptive perfectionism and depression among college students.

H4: Coping styles mediate the relationship between maladaptive perfectionism and depression among college students.

H5: Loneliness mediates the relationship between adaptive perfectionism and depression among college students.

H6: Loneliness mediates the relationship between maladaptive perfectionism and depression among college students.

H7: Self-esteem mediates the relationship between adaptive perfectionism and depression among college students.

H8: Self-esteem mediates the relationship between maladaptive perfectionism and depression among college students.

## 4 Methods

### 4.1 Sample and data collection

This study conducted data collection from January 12 to April 1, 2025, through an anonymous online questionnaire administered via Sojump (www.sojump.com). To ensure a representative sample, a minimum sample size of 1,236 participants was required, based on the calculation formula proposed by Kline (2018) (number of items × 10 + number of items × 10 × 20%). The study targeted students from multiple universities in China and employed a simple random sampling procedure to select participants. Specifically, after obtaining the official student registry from the participating universities, a computer-generated random number procedure was used to select students from the complete list, ensuring that each student had an equal probability of selection. The questionnaire was then distributed in class by teachers through official WeChat accounts, survey links, and QR codes. Participants filled out the questionnaire randomly and voluntarily. Informed consent was given by each participant to ensure the protection of personal privacy and data security. Out of 1,240 collected questionnaires, 1,220 valid responses were retained after eliminating invalid and duplicate entries. The descriptive statistical results of the study are shown in Table 1. Within the sample, 34.2% of participants were male and 65.8% were female. Gender, age, only-child status, and grade level did not have a significant effect on depression levels; however, family income and living situation had a significant impact on depression levels. Further multiple comparisons revealed that depressive symptoms were more prevalent among college students from low-income families than among those from higher-income families, while students living with their parents or only with their fathers were less likely to experience depression compared to students in other living situations.

**Table 1:** Demographic characteristics of the sample.

Demographic characteristic	Category	Quantity	Percentage	Depression (*p*)
Gender	Male	417	34.2%	0.089
	Female	803	65.8%	
Age	≤ 18	239	19.6%	0.825
	19–20	766	62.8%	
	21–22	184	15.1%	
	≥23	31	2.5%	
Only child	Only child	195	16.0%	0.111
	Non-only child	1,025	84.0%	
Grade level	Freshman	557	45.7%	0.109
	Sophomore	528	43.3%	
	Junior	57	4.7%	
	Senior	78	6.4%	
Family monthly income	≤ 1,000	67	5.5%	0.028
	1,000–3,000	206	16.9%	
	3,000–6,000	406	33.3%	
	6,000–10,000	314	25.7%	
	10,000–15,000	132	10.8%	
	15,000–20,000	49	4.0%	
	≥20,000	46	3.8%	
Family situation	Living with both parents	1,025	84.0%	0.001
	Living only with mother (due to parental divorce or father's passing)	67	5.5%	
	Living only with father (due to parental divorce or mother's passing)	22	1.8%	
	Living with grandparents or other relatives	106	8.7%	

### 4.2 Measurement

This study employed the Frost Multidimensional Perfectionism Scale (FMPS) developed by Frost et al. (1990) and translated and revised by Fei and Xu (2006) for the Chinese cultural context, which has been validated for use in China. The scale consists of 27 items across 5 dimensions: Concern over mistakes (6 items), Personal standards (6 items), Parental expectations (5 items), Doubts about action (4 items), and Organization (6 items). The first four dimensions are related to MP, while the Organization dimension is associated with AP. In this study, the Cronbach's α for AP and MP were 0.851 and 0.959, respectively.

This study used the Simplified Coping Style Questionnaire (SCSQ) developed by Xie (1998), tailored for the Chinese population. The scale consists of 20 items, divided into two dimensions: PCS (12 items) and NCS (8 items). In this study, the Cronbach's α for PCS and NCS were 0.949 and 0.900, respectively.

The study also employed the University of California Los Angeles Loneliness Scale (UCLA-LS) developed by Russell (1996), which has shown good reliability and validity in China (Wang et al., 2021). The scale consists of 20 items, with 9 items reverse scored. In this study, the Cronbach's α for the UCLA-LS was 0.974.

The Rosenberg Self-Esteem Scale (RSES), developed by Rosenberg (1965), was used to assess SE. The scale has been shown to have good reliability and validity in China (Wang and Wu, 2022). It consists of 10 items, with 5 items reverse scored. In this study, the Cronbach's α for the RSES was 0.937.

Lastly, the Self-rating Depression Scale (SDS), developed by Zung (1965), was used to assess depression. The scale has been shown to have good reliability and validity in China (Wang et al., 2024). It consists of 20 items, with 10 items reverse scored. In this study, the Cronbach's α for the SDS was 0.970.

In this study, except for the FMPS which uses a 5-point Likert scale (ranging from 1 to 5), all other scales use a 4-point Likert scale (ranging from 1 to 4). For all scales, higher scores correspond to higher levels of the respective variables.

### 4.3 Statistical analysis

The predictive approach known as Partial Least Squares Structural Equation Modeling (PLS-SEM) is quite flexible when dealing with small samples and non-normal data, and it can manage intricate model structures (Kono and Sato, 2023; Cheah et al., 2021). In this study, Smart PLS 4 was used for data analysis. Given that the sample size in this study was 1,220 participants and the model structure involved 5 constructs and 103 observed indicators, which adds a level of complexity, PLS-SEM was chosen as the analysis tool due to its high adaptability and practical value.

## 5 Results

### 5.1 Measurement model

The results, as shown in Table 2, indicate that the skewness values for all variables did not exceed 3, and the kurtosis values were all less than 10, which meets the commonly accepted statistical standards. This suggests that all variables in this study follow a normal distribution and there are no outliers (Kline, 2005). The Kaiser–Meyer–Olkin (KMO) values for all measurement instruments were greater than 0.5, and the *p*-values for Bartlett's Test of Sphericity were all less than 0.05, indicating that the data were suitable for factor analysis.

**Table 2:** Statistical summaries for each variable.

Variables	M ±SD	Minimum	Maximum	Skewness	Kurtosis	KMO	Bartlett (*p*)
AP	21.500 ± 4.047	6.000	30.000	−0.746	1.287	0.850	0.000
MP	49.240 ± 14.022	21.000	96.000	1.063	1.421	0.972	0.000
PCS	35.360 ± 6.957	12.000	48.000	−0.864	0.735	0.959	0.000
NCS	14.600 ± 3.984	8.000	32.000	1.502	3.517	0.935	0.000
Loneliness	41.610 ±15.076	20.000	100.000	0.558	−0.891	0.983	0.000
SE	29.950 ± 7.278	11.000	40.000	−0.639	−0.634	0.936	0.000
Depression	35.660 ± 13.085	20.000	75.000	1.191	0.226	0.983	0.000

M ± SD, Mean ± Standard Deviation; AP, adaptive perfectionism; MP, maladaptive perfectionism; PCS, positive coping style; NCS, negative coping style; SE, self-esteem.

Subsequently, following the advice of Hair et al. (2021), measurement model analysis was then used to assess the scales' accuracy and consistency. Composite Reliability (CR) and the external loadings of the items were the main criteria used to evaluate reliability. The recommendations state that both external loadings and CR must be more than 0.708 (Hair et al., 2021). In this study, as shown in Table 3, except for MP18 and Loneliness 2, the external loadings of the remaining items met the standard, and therefore, MP18 and Loneliness 2 were removed. Furthermore, all constructions' CRs were greater than 0.708, showing strong internal consistency reliability (Hair et al., 2021). The external loadings for all items and the overall level of CR for each construct were high, meeting the reliability requirements. In terms of validity, the HTMT ratio and the Fornell-Larcker criterion were used to test discriminant validity, while Average Variance Extracted (AVE) was used to examine convergent validity. The findings showed that each construct's AVE was higher than 0.5 (Table 3), HTMT coefficients remained under the 0.85 threshold (Table 4), and each construct's AVE square root was higher than its correlations with the remaining constructs (Table 5). These results meet the required standards, demonstrating the model's strong validity (Hair et al., 2021).

**Table 3:** Reliability and validity.

Constructs	Items	Loadings	CR	Cronbach's α	AVE
AP	AP 1	0.725	0.889	0.851	0.573
	AP 2	0.763			
	AP 3	0.766			
	AP 4	0.729			
	AP 5	0.778			
	AP 6	0.778			
MP	MP 1	0.735	0.962	0.959	0.559
	MP 2	0.719			
	MP 3	0.764			
	MP 4	0.735			
	MP 5	0.742			
	MP 6	0.805			
	MP 7	0.795			
	MP 8	0.746			
	MP 9	0.749			
	MP 10	0.748			
	MP 11	0.726			
	MP 12	0.715			
	MP 13	0.796			
	MP 14	0.733			
	MP 15	0.776			
	MP 16	0.759			
	MP 17	0.714			
	MP 19	0.766			
	MP 20	0.712			
	MP 21	0.726			
PCS	PCS 1	0.755	0.955	0.949	0.639
	PCS 2	0.770			
	PCS 3	0.806			
	PCS 4	0.826			
	PCS 5	0.796			
	PCS 6	0.811			
	PCS 7	0.808			
	PCS 8	0.808			
	PCS 9	0.817			
	PCS 10	0.818			
	PCS 11	0.798			
	PCS 12	0.777			
NCS	NCS 1	0.744	0.918	0.900	0.585
	NCS 2	0.796			
	NCS 3	0.782			
	NCS 4	0.799			
	NCS 5	0.758			
	NCS 6	0.780			
	NCS 7	0.727			
	NCS 8	0.729			
Loneliness	Loneliness 1	0.841	0.976	0.974	0.679
	Loneliness 3	0.803			
	Loneliness 4	0.849			
	Loneliness 5	0.854			
	Loneliness 6	0.822			
	Loneliness 7	0.876			
	Loneliness 8	0.813			
	Loneliness 9	0.815			
	Loneliness 10	0.805			
	Loneliness 11	0.870			
	Loneliness 12	0.801			
	Loneliness 13	0.804			
	Loneliness 14	0.821			
	Loneliness 15	0.770			
	Loneliness 16	0.806			
	Loneliness 17	0.762			
	Loneliness 18	0.851			
	Loneliness 19	0.828			
	Loneliness 20	0.844			
SE	SE 1	0.747	0.946	0.937	0.638
	SE 2	0.796			
	SE 3	0.855			
	SE 4	0.788			
	SE 5	0.779			
	SE 6	0.813			
	SE 7	0.833			
	SE 8	0.724			
	SE 9	0.824			
	SE 10	0.817			
Depression	Depression 1	0.762	0.972	0.970	0.634
	Depression 2	0.775			
	Depression 3	0.802			
	Depression 4	0.799			
	Depression 5	0.790			
	Depression 6	0.780			
	Depression 7	0.779			
	Depression 8	0.794			
	Depression 9	0.813			
	Depression 10	0.792			
	Depression 11	0.803			
	Depression 12	0.781			
	Depression 13	0.795			
	Depression 14	0.814			
	Depression 15	0.761			
	Depression 16	0.774			
	Depression 17	0.822			
	Depression 18	0.824			
	Depression 19	0.849			
	Depression 20	0.814			

AP, adaptive perfectionism; MP, maladaptive perfectionism; PCS, positive coping style; NCS, negative coping style; SE, self-esteem.

**Table 4:** Discriminant validity (HTMT criterion).

Variables	AP	Depression	Loneliness	MP	NCS	PCS	SE
AP							
Depression	0.248						
Loneliness	0.171	0.483					
MP	0.162	0.269	0.353				
NCS	0.179	0.287	0.239	0.138			
PCS	0.355	0.441	0.297	0.144	0.432		
SE	0.296	0.639	0.562	0.361	0.310	0.480	

AP, adaptive perfectionism; MP, maladaptive perfectionism; PCS, positive coping style; NCS, negative coping style; SE, self-esteem.

**Table 5:** Discriminant validity (Fornell-Larcker criterion).

Variables	AP	Depression	Loneliness	MP	NCS	PCS	SE
AP	**0.757**						
Depression	−0.231	**0.797**					
Loneliness	−0.158	0.472	**0.824**				
MP	−0.142	0.278	0.360	**0.748**			
NCS	−0.166	0.281	0.236	0.151	**0.765**		
PCS	0.324	−0.428	−0.287	−0.154	−0.402	**0.799**	
SE	0.270	−0.614	−0.541	−0.370	−0.296	0.458	**0.799**

The square root of the AVE shown as the bolded diagonal values in the table; AP, adaptive perfectionism; MP, maladaptive perfectionism; PCS, positive coping style; NCS, negative coping style; SE, self-esteem.

### 5.2 Confirmatory factor analysis

The results of the confirmatory factor analysis (CFA) in this study indicate that after modification, the overall model has good fit. The model demonstrated good fit to the data (χ^2^/df = 1.661; RMSEA = 0.034; GFI = 0.902; NFI = 0.905; CFI = 0.946; IFI = 0.947; TLI = 0.943), all of which satisfied the recommended criteria proposed by Hu and Bentler (1998).

### 5.3 Structural model

This study tested for multicollinearity among the variables in the model. The results show that the Variance Inflation Factors (VIF) are all below 3 (Table 6), demonstrating that this study does not have a significant multicollinearity problem (Gómez et al., 2020).

**Table 6:** VIF.

Variables	AP	Depression	Loneliness	MP	NCS	PCS	SE
AP		1.145	1.021		1.021	1.021	1.021
Depression							
Loneliness		1.487					
MP		1.216	1.021		1.021	1.021	1.021
NCS		1.226					
PCS		1.477					
SE		1.759					

AP, adaptive perfectionism; MP, maladaptive perfectionism; PCS, positive coping style; NCS, negative coping style; SE, self-esteem.

A PLS bootstrapping process with 5,000 subsamples was conducted to assess both the magnitude and significance of the path coefficients (Hair et al., 2021). After controlling for covariates such as family monthly income and family situation, the variables' correlations are displayed in Table 7. Except for AP, MP, and NCS (*p* > 0.05), all other factors were significant predictors of depression. Among these, SE was the strongest determinant (β = −0.415, *p* = 0.000), followed by loneliness (β = 0.178, *p* = 0.000) and PCS (β = −0.155, *p* = 0.000). Therefore, H1 and H2 are not supported.

**Table 7:** Hypothesis testing.

Hypothesis	β	2.50%	97.50%	*t*	*p*	Results
AP → Depression	−0.030	−0.083	0.024	1.089	0.276	Not supported
AP → Loneliness	−0.109	−0.170	−0.052	3.626	0.000	Supported
AP → NCS	−0.148	−0.228	−0.069	3.654	0.000	Supported
AP → PCS	0.308	0.234	0.379	8.358	0.000	Supported
AP → SE	0.221	0.164	0.280	7.529	0.000	Supported
Loneliness → Depression	0.178	0.112	0.245	5.183	0.000	Supported
MP → Depression	0.025	−0.032	0.079	0.874	0.382	Not supported
MP → Loneliness	0.344	0.294	0.400	12.748	0.000	Supported
MP → NCS	0.130	0.063	0.204	3.616	0.000	Supported
MP → PCS	−0.111	−0.175	−0.053	3.482	0.001	Supported
MP → SE	−0.339	−0.392	−0.288	12.710	0.000	Supported
NCS → Depression	0.044	−0.011	0.103	1.522	0.128	Not supported
PCS → Depression	−0.155	−0.218	−0.090	4.798	0.000	Supported
SE → Depression	−0.415	−0.488	−0.340	11.006	0.000	Supported

AP, adaptive perfectionism; MP, maladaptive perfectionism; PCS, positive coping style; NCS, negative coping style; SE, self-esteem.

Finally, the model's explanatory power (*R*^2^), adjusted *R*^2^, and predictive relevance (Stone-Geisser's *Q*^2^) were tested. The *R*^2^ value for depression was 0.433, and the adjusted *R*^2^ value was 0.430, indicating that the predictors explain 43.0% of the total variance in depression. Furthermore, the *Q*^2^ value for depression was 0.270, which is greater than 0, suggesting that the empirical model has good predictive relevance (Hair et al., 2021).

### 5.4 Mediation effect analysis

Table 8 displays the findings of the mediation effect analysis. In the total effect analysis, AP had a significant negative effect on depression (β = −0.195, *p* = 0.000), while MP had a significant positive effect on depression (β = 0.250, *p* = 0.000). Additionally, further indirect effect analysis revealed that, in the relationship between AP and depression, SE had the strongest mediating effect, accounting for 47.179% of the total effect. This was followed by PCS, which accounted for 24.615%, and loneliness, which accounted for the smallest portion at 9.744%. In the relationship between MP and depression, SE still had the strongest mediating effect, accounting for 56.400%, followed by loneliness, accounting for 24.400%, with PCS accounting for the smallest portion at 6.800%. Therefore, H5, H6, H7, and H8 are supported, while H3 and H4 are partially supported.

**Table 8:** Mediation effect test.

Effect type	Path	β	*t*	*p*	2.5%	97.50%	Percentage
Total effect	AP → Depression	−0.195	5.612	0.000	−0.264	−0.128	100%
Total indirect effect	AP → Depression	−0.165	7.549	0.000	−0.211	−0.125	84.615%
Specific indirect effects	AP → PCS → Depression	−0.048	3.980	0.000	−0.072	−0.025	24.615%
	AP → Loneliness → Depression	−0.019	2.827	0.005	−0.034	−0.008	9.744%
	AP → SE → Depression	−0.092	6.188	0.000	−0.123	−0.065	47.179%
Total effect	MP → Depression	0.250	8.283	0.000	0.193	0.309	100%
Total indirect effect	MP → Depression	0.225	11.570	0.000	0.189	0.265	90.000%
Specific indirect effects	MP → PCS → Depression	0.017	2.852	0.004	0.007	0.030	6.800%
	MP → Loneliness → Depression	0.061	4.842	0.000	0.038	0.087	24.400%
	MP → SE → Depression	0.141	8.419	0.000	0.110	0.175	56.400%

AP, adaptive perfectionism; MP, maladaptive perfectionism; PCS, positive coping style; NCS, negative coping style; SE, self-esteem.

## 6 Discussion

This study found that neither AP nor MP significantly predicted depression among college students, which did not support H1 and H2. This result is inconsistent with existing research, which suggests that AP can protect college students from the negative impact of depression, while MP is more likely to lead to depression (Fang and Wang, 2024; Olmedilla et al., 2022). This discrepancy may be due to the fact that the perfectionism traits of college students have not yet formed stable behavioral and emotional patterns. College students, in a transitional developmental period, may experience significant fluctuations in their perfectionistic tendencies as they adapt to environmental changes when facing external pressure. Therefore, the direct effect of perfectionism on depression may fluctuate, as it is more influenced by specific situational factors and individual coping resources. As a result, after controlling for mediating variables, the direct predictive relationship between perfectionism and depression was not found to be significant. Furthermore, this study used Chinese college students as the sample. In Chinese culture, diligence and the pursuit of excellence are widely recognized. High standards and self-expectations may not only be seen as positive traits but also as normal social expectations or self-motivation for students with AP (Jia et al., 2025). Moreover, influenced by Confucian ideals of self-improvement and self-discipline, college students may interpret traits of MP, such as sensitivity to mistakes and concern about others' evaluations, as manifestations of caution and self-discipline rather than merely negative characteristics (Suh et al., 2023). This cultural understanding may weaken the direct negative impact of perfectionism on depression, which could explain the lack of significant direct effects observed in this study. In addition, the non-significant direct effects may be partly related to the way perfectionism was defined and measured in this study. Using the organization dimension of the FMPS as the indicator of AP may not fully capture its broader conceptual meaning. Some scholars have suggested that Personal Standards may also reflect adaptive characteristics (Stoeber and Otto, 2006). Different dimensional classifications may therefore influence the observed associations among variables.

The study revealed that PCS mediates the relationship between both AP and depression, and MP and depression, while NCS did not mediate these relationships. This result partially supports H3 and H4. It aligns with existing research to some extent, indicating that AP can effectively alleviate college students' depressive emotions through PCS (Dong et al., 2024; Samfira and Palos, 2021), while MP can exacerbate depression through NCS (Montano, 2025). This may be because, under the multiple pressures of academics, interpersonal relationships, and career development, individuals with MP are more prone to self-criticism and heightened sensitivity to failure (Huang et al., 2022). However, this psychological tendency manifests more as internal self-blame and emotional exhaustion, rather than through overt negative avoidance behaviors in response to external pressures (Huang et al., 2022). Therefore, NCS did not serve as a significant mediator in this relationship. Moreover, the study further found that MP indirectly exacerbates depression by weakening PCS. College students are in a critical stage of identity formation and social adaptation, and PCS are essential for their mental health (Lu et al., 2024). However, maladaptive perfectionists tend to be overly sensitive to mistakes, excessively concerned with others' evaluations, and engage in self-neglect, which may weaken their ability to regulate emotions and solve problems. In a highly competitive environment, individuals are more susceptible to emotional distress, thus intensifying depressive responses (Suh et al., 2024).

This study revealed that loneliness mediates the relationship between both AP and depression, and MP and depression, supporting H5 and H6. This result is consistent with existing research, which suggests that AP can alleviate depression by reducing loneliness, while MP exacerbates depression through loneliness (Hee and Hyun, 2023; Visvalingam et al., 2024; Niels-Kessels et al., 2025). College students are in a critical stage of self-identity development and interpersonal relationship formation. Adaptive perfectionists typically have positive self-cognition and high social interaction motivation, and they tend to handle interpersonal relationships constructively. When faced with social setbacks, they are better at regulating their emotions, leading to less loneliness and, consequently, a reduced risk of depression (He and Ding, 2025). In contrast, maladaptive perfectionists, due to their heightened concern for others' evaluations, fear of failure, and self-neglect, are more prone to social anxiety and alienation, which increases loneliness and exacerbates depressive emotions (Blynova et al., 2021). Furthermore, loneliness not only reflects the lack of social support but also symbolizes the depletion of an individual's psychological resources (Hu et al., 2023). For college students, AP helps mobilize both internal and external resources, such as seeking help and actively participating in clubs or social activities, which alleviates loneliness (Rnic et al., 2021). On the other hand, maladaptive perfectionists, influenced by negative cognitive tendencies, are more likely to fall into self-neglect and feelings of helplessness in the experience of loneliness, ultimately leading to an increase in depressive symptoms (Rnic et al., 2021).

This study revealed that SE mediates the relationship between both AP and depression, and MP and depression, supporting H7 and H8. This result is consistent with existing research, which indicates that AP can alleviate depression through SE, while MP exacerbates depression by weakening SE (Chai et al., 2020). On one hand, the college stage is an important period for individuals' self-identity and social role transitions. AP promotes positive adaptation in academic, interpersonal, and career development by enhancing SE through stable self-worth recognition, thereby reducing the risk of depression (Raedeke et al., 2021). In contrast, MP, by reinforcing the focus on personal shortcomings and external evaluations, weakens SE and increases difficulties in adaptation, contributing to depressive tendencies (Doyle and Catling, 2022). On the other hand, SE plays a key role in regulating individuals' emotional responses and cognitive evaluations (Burkitt, 2024). AP helps enhance SE by fostering self-affirmation, which in turn supports the development of stable emotional coping mechanisms, thus lowering the risk of depression (Jiang and Zhang, 2023; Raedeke et al., 2021). In contrast, individuals with MP, due to prolonged self-neglect and catastrophic thinking, weaken their SE, increase feelings of helplessness, and exacerbate depressive symptoms (Lei et al., 2024; Fearn et al., 2022).

## 7 Implications and limitations

### 7.1 Implications

The findings of this study contribute to the theoretical development of research on college students' mental health. On one hand, based on Social Cognitive Theory and the Stress-Vulnerability Model, this study is the first to simultaneously introduce coping styles, loneliness, and SE, constructing a parallel mediation model that reveals the comprehensive process through which adaptive and MP influences depression via multiple psychological mechanisms. The study broadens existing theoretical perspectives on how perfectionism relates to mental health outcomes. On the other hand, this study methodically demonstrates how internal resources play a part in the connection between depression and perfectionism from three dimensions: cognitive-behavioral responses (coping styles), social-emotional experiences (loneliness), and self-system cognition (SE). It supports the Stress-Vulnerability Model's hypothesis that internal vulnerability factors amplify negative effects, providing new empirical evidence for refining and deepening the Stress-Vulnerability Theory. Additionally, this study found that the two different dimensions of perfectionism may not directly influence depression among college students in a specific cultural context. This finding challenges the view that MP is a core vulnerability factor for depression, offering a new perspective for research in the field of psychology. Interestingly, this study also found that gender, age, only-child status, and grade level did not significantly influence depression levels, while family income and living situation had significant effects. Students from low-income families were more likely to experience depression, while students living with their parents or fathers had a lower risk of depression. This further supports the theoretical assumption that the family environment is an important source of social support, providing empirical evidence for considering socio-economic background in future mental health interventions.

The practical importance of this study lies in providing specific guidance for mental health interventions for college students. The findings imply that interventions should be customized to different types of perfectionism. For adaptive perfectionists, the focus should be on helping them set reasonable goals while maintaining self-motivation, avoiding internal conflict caused by excessively high self-expectations. Universities can offer goal decomposition training and time management guidance to help students break long-term goals into achievable, phased tasks, balancing academic and life pressures. For maladaptive perfectionists, the focus should be on correcting negative cognition and cultivating self-acceptance. This can be achieved by guiding students to reflect on failures through journaling, reducing catastrophic thinking about mistakes, and using peer modeling to help them learn healthier ways of coping with failure and shifting their thought patterns. In addition, SE, PCS, and loneliness function as full intermediaries in the relationship between different aspects of perfectionism and depression. Therefore, universities can promote SE by offering self-identity education, self-worth exploration workshops, and strengthening positive feedback mechanisms to help students build a stable self-evaluation system; by cognitive-behavioral training, stress management courses, and emotional regulation group counseling help students master effective coping strategies; by building an interactive campus environment, offering social courses, and establishing peer support systems enhance students' sense of social belonging and reduce loneliness, effectively preventing depression. Finally, considering the emphasis on high standards and external evaluation in Chinese culture, along with the influence of the family environment on students' mental health, mental health education should help college students develop a more autonomous and resilient sense of self-worth while acknowledging social expectations. This approach will promote their psychological development toward individualization within a collectivist culture. Special attention should be given to students from low-income families by providing more psychological support and resources to reduce the negative impact of financial pressures. At the same time, family members, particularly parents, should actively engage in students' mental health development, strengthen the family support system, and promote students' emotional stability and psychological resilience.

### 7.2 Limitations and future research directions

Firstly, this study relied on self-report questionnaires for data collection. While this method is convenient and suitable for large sample surveys, it may be influenced by participants' understanding biases and response styles. Future research could incorporate qualitative interviews or focus group discussions to further supplement and enrich the data sources. Secondly, this study employed a cross-sectional design, making it difficult to accurately infer causal relationships between variables. Future research could use longitudinal designs to track the dynamic changes in perfectionism, coping styles, loneliness, SE, and depression among college students, providing a clearer understanding of the causal relationships. Third, regarding the measurement and dimensional classification of perfectionism, this study defined the organization dimension of the FMPS as the indicator of AP, which may limit a comprehensive understanding of the construct. Future research could compare alternative dimensional classifications, such as incorporating the personal standards dimension into the AP framework, to more accurately examine the associations between different facets and mental health variables. Lastly, although this study considered the influence of the family environment, it did not explore in depth the impact of factors such as family structure and family functioning on college students' mental health. Future studies could examine multiple factors of family background, including parental education levels and family support systems, to better understand the role of the family environment in mental health.

## 8 Conclusion

This study thoroughly explored the mechanisms through which adaptive and MP influence depression among college students, highlighting the important role of SE, coping styles, and loneliness as mediating variables. The results of the mediation effect analysis showed that although AP significantly negatively predicts depression and MP has a significant positive effect at the total effect level, these effects mainly occur through indirect pathways. Specifically, SE serves as the primary mediator between both dimensions of perfectionism and depression. PCS and loneliness play secondary roles in different pathways, reflecting the differences and hierarchical nature of psychological mechanisms in the influence process. This finding not only reveals the multi-pathways through which perfectionism affects depression, but also suggests that future mental health interventions should target different types of perfectionism, focusing on enhancing college students' SE and adaptability, and reducing feelings of loneliness to achieve precise and targeted interventions. This research sheds new light on how individual differences relate to mental health, offering theoretical support for university psychological education and intervention practices.

## Data Availability

The datasets presented in this study can be found in online repositories. The names of the repository/repositories and accession number(s) can be found in the article/supplementary material.
